# Supplementary material for: Rethinking risk prediction: The role of albumin and other parameters in implant-associated complications after hip or knee arthroplasty
Source: PLoS One. 2024 Jul 29;19(7):e0306468. doi: 10.1371/journal.pone.0306468 (PMC11285930; doi:10.1371/journal.pone.0306468)
Supplement: Attachment — Submitted filename: Response to Reviewers.docx [file pone.0306468.s002.docx]

Hradec Králové, 01/05/2024

Dear Editor-in-Chief, dear editors

We gratefully acknowledge the editor and reviewers for their valuable comments on our manuscript entitled “Rethinking risk prediction: The role of albumin and other parameters in implant-associated complications after hip or knee arthroplasty”. We have thoroughly considered all the suggestions and recommendations provided and have incorporated them into our revised version of the manuscript. Reviewers’ commentaries regarding statistical analysis were consulted and approved by a statistician, who also contributed to the relevant responses. Moreover, the tables were revised (i.e. NRI values in Table 3), and the other sections have been condensed to emphasize the key point better.  Furthermore, we have included all the requirements stated by the Academic editor:

**Requirement 1:** Please ensure that your manuscript meets PLOS ONE's style requirements, including those for file naming. The PLOS ONE style templates can be found at https://journals.plos.org/plosone/s/file?id=wjVg/PLOSOne_formatting_sample_main_body.pdf and <https://journals.plos.org/plosone/s/file?id=ba62/PLOSOne_formatting_sample_title_authors_affiliations.pdf>

**Authors´ comment:** We have checked the revised manuscript according to PLOS ONE’s style requirements.

**Requirement 2:** Thank you for stating the following financial disclosure:

"Petr Domecky as a PhD student is supported by Charles University (Project SVV 260 665)."

Please state what role the funders took in the study. If the funders had no role, please state: ""The funders had no role in study design, data collection and analysis, decision to publish, or preparation of the manuscript."" If this statement is not correct you must amend it as needed. Please include this amended Role of Funder statement in your cover letter; we will change the online submission form on your behalf.

**Authors´ comment:** The funders had no role in study design, data collection and analysis, decision to publish, or preparation of the manuscript.

**Requirement 3:** In the online submission form, you indicated that [The data that support the findings of this study are available from University Hospital Hradec Kralove but restrictions apply to the availability of these data, which were used under license for the current study, and so are not publicly available. Data are however available from the authors upon reasonable request and with permission of University Hospital Hradec Kralove.]

All PLOS journals now require all data underlying the findings described in their manuscript to be freely available to other researchers, either 1. In a public repository, 2. Within the manuscript itself, or 3. Uploaded as supplementary information.

This policy applies to all data except where public deposition would breach compliance with the protocol approved by your research ethics board. If your data cannot be made publicly available for ethical or legal reasons (e.g., public availability would compromise patient privacy), please explain your reasons on resubmission and your exemption request will be escalated for approval.

**Authors´ comment:**  After consultation with the department where the data collection took place and after careful consideration by the Ethics Committee, the data in anonymised form, including statistical outputs, are attached as a supplement.

Data Availability: All relevant data are within the manuscript and its Supporting Information files.

**Requirement 4:** We note you have included a table to which you do not refer in the text of your manuscript. Please ensure that you refer to Table 1 and 2 in your text; if accepted, production will need this reference to link the reader to the Table.

**Authors´ comment:** Thank you for this commentary. We have updated all the cross-references.

**Requirement 5:** Please review your reference list to ensure that it is complete and correct. If you have cited papers that have been retracted, please include the rationale for doing so in the manuscript text, or remove these references and replace them with relevant current references. Any changes to the reference list should be mentioned in the rebuttal letter that accompanies your revised manuscript. If you need to cite a retracted article, indicate the article’s retracted status in the References list and also include a citation and full reference for the retraction notice.

**Authors´ comment:** We have checked all the references. No retracted articles are included in our manuscript.

We are highly thankful for the time and effort invested in reviewing our work, and we believe that the comments have significantly improved the quality of our research.

Kind regards

Josef Maly

Department of Social and Clinical Pharmacy

Faculty of Pharmacy in Hradec Kralove, Charles University

Ak. Heyrovskeho 1203/8, 500 05 Hradec Kralove, Czech Republic

___________________________________________________________________________

**Reviewer: 1**

**Comments to the Author:** The aims and objectives of the study is apt, the methodology including the design, data collection, the outcomes and statistical analysis is good. The ethical approval and consent were duly taken. The result was properly analysed.

Authors´ comment: We greatly appreciated your positive feedback.

Changes in the text: None.

**Reviewer: 2**

**Comments to the Author:** I have been given the opportunity to review your article aimed at predicting complications associated with total hip (THA) and knee (TKA) arthroplasty, highlighting the high risk of post-operative inflammatory reactions. The prospective observational study conducted on patients with primary knee or hip osteoarthritis undergoing THA or TKA was described with absolute clarity, identifying albumin, prealbumin, Intensive Care Infection Score (ICIS), Nutritional Risk Index, and white blood cell count as potential predictors. However, it should be noted that prosthetic infection may also result from the complex concomitant action of multiple factors: bacteria, prostheses, and host weakness. In this regard, I would kindly ask you to briefly elaborate on the possible role of host-related concomitant factors, as it is known that the immune response is able to limit infection. I would like to bring to your attention, solely for illustrative purposes, an interesting study (DOI: 10.1186/S10195-021-00607-6) that may be of specific interest to you and could potentially be considered in your references, if deemed useful. This study highlights that among the intraoperative reasons for the development of infectious complications after joint arthroplasty are a duration of surgery exceeding 180 minutes, significant blood loss (more than 800 ml), blood transfusion, excessive tissue trauma, the presence of nosocomial bacterial strains, and failure to adhere to aseptic and antiseptic rules.

Authors´ comment: Thank you for this suggestion. In the Discussion section, we have elaborated on the possible role of host-related concomitant factors.  Furthermore, we have also included the comparison between the possible role of host-related concomitant factors and intraoperative reasons, which were available for our population (see new Table 2).

Changes in the text: A new part added to the discussion section:

Line 255–264: Other factors could influence the risk of IAC after TKA or THA, as suggested in the article by Basile et al. [25] Among the malnutrition, which we also included in our study as NRI or albumin/prealbumin, intraoperative factors, such as patient-specific factors (e.g. obesity, systemic infections) and surgical conditions (e.g. prolonged surgery, significant blood loss, alongside adherence to aseptic techniques and postoperative care) should be taken into account as risk factors for IAC. Due to this, we have also compared the baseline characteristics of the group with IAC and the group without IAC, as stated in Table 2. Adherence to the aseptic techniques and postoperative care were no issue, as the university hospital had internal preoperative, perioperative and postoperative standards. Each patient received the same internal standard for preoperative care, and also the same internal standard was used during and after the surgery.

Page 9: a new Table 2 has been added: Comparison of population characteristics between patients according to IAC status.

**Comments to the Author:** Your study clearly demonstrates that parameters such as albumin, NRI, prealbumin, white blood cells, and ICIS can predict implant-related complications, emphasizing the importance of comprehensive inflammatory and nutritional assessments in managing and predicting complications, with significant implications for patient risk assessment. The text discusses the results of the study and refers to previous studies to provide context and comparisons. However, some points may require clarification. In this regard, it is necessary to clarify whether specific measures were taken to minimize the risk of confounding in data collection and statistical analysis. The text mentions some limitations of the study, such as the small sample size and the short follow-up of patients. However, it may be useful to further explore how these limitations may have influenced the results and conclusions of the study. It would be helpful to discuss more thoroughly the clinical implications of the study results and how they may affect daily clinical practice. In conclusion, the analysis you have drawn provides a good overview of the study's objectives, methods, results, and conclusions, although some parts could be improved with additional clarification and insights, as mentioned above.

Authors´ comment: Thank you for your comment. We have included information regarding the limitations of the study. About whether specific measures were taken to minimize the risk of confounding in data collection and statistical analysis, we should clarify that we carefully defined inclusion and exclusion criteria to ensure a homogeneous study population even with consecutive samples. Moreover, we could not perform a multivariate analysis (see Reviewer 3 comments), but we compared groups according to the IAC status. No differences were observed (see new Table 2). The conclusion was also edited to include the daily practice recommendations. However, the results might be limited, mainly due to the points in the limitations sections. Furthermore, it should be noted that this response was consulted with a statistician.

Changes in the text: Limitation and conclusion section has been edited:

Line 276–278: Therefore, the results are only for the early-term IAC (6–7 weeks as stated in the methodology section) and not for the long-term IAC.

Line 282–283: Nevertheless, we analysed both populations regarding their IAC status, and there were no differences. Therefore, this is not a limitation.

Line 293–294: Notably, albumin levels show significant preoperative differences related to IAC status **and patients with low preoperative albumin levels might benefit from the proper nutritional support to prevent the negative outcomes.**

Line 298–302: Incorporating other malnutrition or inflammatory parameters among standard perioperative examinations might provide superior outcomes and allow monitoring of patients at higher risk for negative outcomes. Further research with a larger cohort of patients is warranted to substantiate these observations, especially their clinical significance.

**Reviewer: 3**

**Comments to the Authors:**

The rationale for selecting the specific inflammatory and malnutrition parameters should be better justified in the background. While the authors mention that CRP is the most commonly used parameter, it is unclear why the other parameters were chosen.

Authors´ comment: According to the current practice, the CRP is the most commonly used parameter. Regarding its limitations related to its pharmacokinetics, the rationale was to understand how the other parameters work and if they can provide better results. The background has been slightly edited to include the rationale.

Changes in the text:

Line 57–58: Therefore, the neutrophile-lymphocyte (NLR) count could signify underlying complications not immediately evident through CRP levels alone

Line 58–61: Furthermore, malnutrition has proven to be a valuable predictor of IAC, **therefore, there might be a need for these parameters such as The Prognostic Inflammatory and Nutritional Index (PINI) and Nutritional Risk Index (NRI), which allows for a more comprehensive assessment of patients’ status.**

Line 66–75: Its application to the orthopaedics field is innovative, aiming to ascertain whether it can offer additional predictive value beyond the capabilities of CRP or the other parameters (NLR, PINI, NLR). Given the limitations of CRP as a standalone parameter to predict the IAC for the abovementioned reasons, we have hypothesized that various malnutrition or inflammatory parameters might offer a superior prediction of IAC during the preoperative and postoperative courses, and these parameters included ICIS and PINI that have not yet been investigated in orthopaedics. If these parameters prove reliable in predicting IAC, it will allow better identification of at-risk patients, leading to better pre- and postoperative care optimisation. A parameter meeting all of the requirements would significantly benefit clinical practice as it could reduce IAC and improve overall patient outcomes.

**Comments to the Authors:**

The sample size calculation is not provided so if it's possible it's better include it.

Authors´ comment: As this is a pivotal study, the sample size was not predetermined. The initial documentation, which included study design, hypothesis formulation, and study protocol, was prepared in 2018. We operated under the assumption that there might be additional suitable parameters beyond C-reactive protein (CRP) for evaluating IAC. Unfortunately, the pivotal studies examining individual parameters were conducted after our initial preparatory work or did not include analysis of the Receiver Operating Characteristic (ROC) curve with Area Under the Curve (AUC) estimation to precisely define the null hypothesis value (i.e. AUC for null hypothesis):

- Yombi et al. (2016) did not include ROC analysis and focused only on TKA with the neutrophil-to-lymphocyte ratio.
- Zhao et al. (2020) included ROC analysis only for the neutrophil-to-lymphocyte ratio.
- Blevins et al. (2018) provided ROC analysis merely for albumin.
- Yuwen et al. (2017) did not perform ROC analysis and studied only albumin.
- Fang et al. (2022) lacked ROC analysis and focused on the nutritional risk index.

Given the insufficient data on the AUC for various parameters, we could not accurately calculate the sample size for our study. Therefore, we have decided to conduct a consecutive diagnostic accuracy study with a limited period. The primary goal was to publish these findings and conduct a follow-up study on the inflammatory parameters of interest. Unfortunately, our study was impeded by the COVID-19 pandemic, resulting in delayed evaluation of the final results. However, we tried to calculate an approximate sample size based on the expected prevalence of IAC. We assumed the AUC we want to achieve for most parameters (i.e. alternative hypothesis = AUC: 0.6-0.7). The calculation was followed with the settings: Type I error (Alpha, significance): 0.05, type II error (Beta, 1-power): 0.20 and AUC ROC: > 0.6 aimed for the 171 patients and 19 outcomes. This calculation was used only to illustrate how many patients we need in consecutive recruitment. Furthermore, it should be noted that this response was consulted with a statistician.

Changes in the text: None.

**Comments to the Authors:** The authors could provide more details on the surgical procedures and postoperative care in the methods if it possible. This information is important I think to understand potential confounding factors and assess the generalizability of the study findings.

Authors´ comment: Thank you for this commentary. We have included this part into the methodology section.

Changes in the text: The new subsection has been added: Preoperative care; Surgical approach and postoperative care.

Line 94–102: Preoperative care

For antibiotic prophylaxis, cefazolin was used; vancomycin was administered in case an allergy to beta-lactams was present. According to the hospital’s internal standards, cefazolin should be administered 30 minutes before surgery at a 2-3 g dose according to body weight. The second dose of cefazolin was given 4 hours after the start of surgery and the next two after 6 hours; in total, four doses were administered. Vancomycin was given at 1-1.5 g according to body weight for two doses 12 hours apart. According to the internal hospital standards, nadroparin or enoxaparin was administered as thrombosis prophylaxis, and patients were switched to rivaroxaban after the fourth day.

Line 103–109: Surgical approach and postoperative care

All THAs were performed using the anterolateral surgical approach. All TKAs were performed using the anteromedial surgical approach. From the first postoperative day, rehabilitation, verticalization, walking with crutches training, and limb loading were indicated according to the surgeon’s decision. Redon drain was extracted according to the amount of fluid drained on the first or second postoperative day, plus standard analgesic and adequate wound care was administered according to the patient's condition. Sutures were removed on day 10.

**Comments to the Authors:** The authors mention that 12 patients developed implant-associated complications. More details on the management, etiology, of complications and their severity could be provided.

Authors´ comment: The details of the IAC have been edited in the relevant part of the results and discussion section.

Changes in the text: More details on the remaining IAC have been added:

Page 8: Table 1: Basic characteristics of patients, surgery, antibiotic prophylaxis and complications

Line 209–213: Mitigated urinary tract infections occurred in two patients (1.26%) and were treated using susceptible antibiotics without a negative impact on arthroplasty. Three (1.89%) patients suffered from thromboembolism after TKA or THA and were managed according to the internal hospital guidelines. One (0.63 %) patient with peroneal nerve paralysis after THA was taken in the care of a physiotherapist and underwent appropriate procedures.

**Comments to the Authors:** While the study identified several potential predictors of IAC, it is unclear how these findings can be used in clinical practice to improve patient outcomes and how. The authors could discuss a little bit in depth the clinical significance of their findings.

Authors´ comment: Thank you for this suggestion. The discussion, limitations and conclusion sections have been edited according to this commentary.

Changes in the text:

Line 201–202: This approach could provide more personalised risk stratification to prevent negative outcomes after THA or TKA.

Line 255–264: Other factors could influence the risk of IAC after TKA or THA, as suggested in the article by Basile et al. [25] Among the malnutrition, which we also included in our study as NRI or albumin/prealbumin, intraoperative factors, such as patient-specific factors (e.g. obesity, systemic infections) and surgical conditions (e.g. prolonged surgery, significant blood loss, alongside adherence to aseptic techniques and postoperative care) should be taken into account as risk factors for IAC. Due to this, we have also compared the baseline characteristics of the group with IAC and the group without IAC, as stated in Table 2. Adherence to the aseptic techniques and postoperative care were no issue, as the university hospital had internal preoperative, perioperative and postoperative standards. Each patient received the same internal standard for preoperative care, and also the same internal standard was used during and after the surgery.

Line 276–278: Therefore, the results are only for the early-term IAC (6–7 weeks as stated in the methodology section) and not for the long-term IAC.

Line 282–283: Nevertheless, we analysed both populations regarding their IAC status, and there were no differences. Therefore, this is not a limitation.

Line 292–294: Notably, albumin levels show significant preoperative differences related to IAC status, suggesting that **patients with low preoperative albumin levels might benefit from the proper nutritional support to prevent the negative outcomes.**

Line 298–302: Incorporating other malnutrition or inflammatory parameters among standard perioperative examinations might provide superior outcomes and allow monitoring of patients at higher risk for negative outcomes. Further research with a larger cohort of patients is warranted to substantiate these observations, especially their clinical significance.

**Comments to the Authors**: The manuscript would benefit from a second round copyediting to improve the grammar, punctuation, and academic style, nut over all was written in very good English.

Authors´ comment: Thank you for this suggestion. The manuscript was double-checked for grammar, punctuation and medical writing. gr

Changes in the text: Grammar, punctuation and medical writing were corrected accordingly. These changes are not highlighted in a revised manuscript as they were cosmetic only.

**Comment to the Authors:** The authors could provide more details on the patient population, including more like comorbidities, and medications if it's possible.

Authors´ comment: Thank you for this suggestion. We have included details of other baseline characteristics in Table 1. Moreover, we have also added the comparison of relevant parameters between the IAC-negative and IAC-positive groups (see new Table 2).

Changes in the text:

Page 8: Table 1: Basic characteristics of patients, surgery, antibiotic prophylaxis and complications

Page 9: Table 2: Comparison of population characteristics between patients according to IAC status

Line 161–162: A comparison between basic characteristics according to the IAC status is presented in Table 2. No differences were observed.

**Comments to the Author:** The authors could provide a little more details on the data collection and analysis methods, including how missing data were handled.

Authors´ comment: More details have been added into the methods section.

Changes in the text: relevant part in the methodology section has been edited appropriately:

Line 138–141: When the observed parameters were not determined during outpatient follow-up, these data were still included in the analyses as missing values (i.e. if CRP was not recorded in a patient, the patient was still included in other analyses for different parameters). None of the patients got missing values for all of the observed parameters in outpatient follow-up

**Comments to the Authors:** The authors could provide more details on the statistical analysis, there are any multiple linear regression analyses that you did analyzing the datas? If no, why you did not performed them?

Authors´ comment: Thank you for this comment. We evaluated the results using multivariate analysis to separate better the individual covariates that can be used to predict IAC (selected cut-offs for individual parameters, selected baseline clinical and demographic characteristics). However, our study faced challenges related to data imbalance, with only 12 IAC cases among 159 patients. The conventional rule of thumb suggests a need for 10-20 observations per independent variable in multivariate analysis (assuming logistic regression due to the binary outcome). Although our sample size initially appeared adequate, the imbalance significantly increased the risk of model overfitting.

Despite these concerns, we have tried to conduct a multivariate logistic analysis (prediction of the negative/positive results based on our data). The results indicated only a moderate correlation between the predictors and the outcome variable (R < 0.3), with the predictors explaining approximately 5% of the variability in IAC. An ANOVA for the model suggested that it might not be suitable for predicting complications. Given these parameters, we concluded that publishing the results would be inappropriate due to the high risk of overfitting. Moreover, the model had wide confidence intervals; therefore, a lower probability of the correct results was observed.

Furthermore, to minimize the potential influence of individual covariates, we have prepared the new Table 2: Comparison of population characteristics between patients according to the IAC using Mann-Whitney U test for continuous variable and Fischer exact test for Categorical variable. Based on this comparison, no differences were observed between the study groups. It should be noted that this response was consulted with a statistician.

Changes in the text: We have updated the statistical analysis section and made a new Table 2 with comparison between patients according to the IAC status:

Line 138–141: When the observed parameters were not determined during outpatient follow-up, these data were still included in the analyses as missing values (i.e. if CRP was not recorded in a patient, the patient was still included in other analyses for different parameters). None of the patients got missing values for all of the observed parameters in outpatient follow-up.

Line 144: Furthermore, Fisher's exact test was used for categorical variables.

Page 9: Table 2: Comparison of population characteristics between patients according to IAC status.

**Comments to the Authors:** You cited KL score referring to Kohn et all. in the (16.) I think it's better refer to the original classification Kellgren JH, Lawrence JS. The Epidemiology of Chronic Rheumatism. Atlas of Standard Radiographs. Vol 2. Oxford, UK: Blackwell Scientific; 1963.
why it's important for you the grade of arthrosis, did you consider it as factor for ICA in your analysis, or it was not influent?

Authors´ comment: Thank you for this insight. We have revised the reference as you suggested. The grade of arthrosis was not primarily considered as a factor of IAC. Nevertheless, the indication for arthroplasty was arthrosis grade III or grade IV. The initial inflammatory irritation in arthrosis with synovitis may or may not affect the initial inflammatory parameters. However, after surgery, the inflammatory parameters are not influenced by the grade of arthrosis. In general, a worse degree of arthrosis means more mobility limitation, pain, limping, etc. Therefore, patients with a higher grade of arthrosis benefit more from the arthroplasty.

Changes in the text: References number 16 has been updated.

**Reviewer 4**

**Comments to the Authors:** The hypothesis of the study was not stated and it is unclear how the authors had chosen the potential predictors. This creates confusion when interpreting the results. For example, we do not expect albumin or nutritional status to drastically fluctuate from one day before surgery to two days post surgery; are the authors using this predictor to inform the pre-operative risk of infection in patients? WCC, ICIS, NLR and CRP on the other hand are more commonly used as diagnostic aid post-operatively. Note that CRP also peaks on post-operative day 2-3, and while it is commonly used to mark a trend, it is not a good benchmark for comparison with potential predictors at this particular time point.

Authors´ comment: Thank you for this commentary. Even though CRP is not a great predictive marker for IAC in terms of its pharmacokinetics, it is still considered the gold standard, as stated in the introduction section. We analyzed the absolute values of the parameter over time. Primarily, we were not looking for a trend but for cut-off values with predictive potential. However, we operated under the assumption that additional suitable parameters might exist beyond CRP to evaluate IAC. Unfortunately, the pivotal studies examining individual parameters were conducted after our initial preparatory work or did not include analysis of the Receiver Operating Characteristic (ROC) curve with Area Under the Curve (AUC) estimation to precisely define the null hypothesis value (i.e. AUC for null hypothesis) (see Reviewer 3 commentary – sample size). Therefore, we have decided to conduct a consecutive diagnostic accuracy study with a limited period. The primary goal was to publish these findings and conduct a follow-up study on the inflammatory parameters of interest. Furthermore, we do not agree that albumin or nutritional status do not drastically fluctuate from one day before surgery to two days post surgery (see for example the following reference: <https://www.ncbi.nlm.nih.gov/pmc/articles/PMC4736779/> or <https://pubmed.ncbi.nlm.nih.gov/28391235/>)

Changes in the text:

Line 57–58: Therefore, the neutrophile-lymphocyte (NLR) count could signify underlying complications not immediately evident through CRP levels alone

Line 58–61: Furthermore, malnutrition has proven to be a valuable predictor of IAC, **therefore, there might be a need for these parameters such as The Prognostic Inflammatory and Nutritional Index (PINI) and Nutritional Risk Index (NRI), which allows for a more comprehensive assessment of patients’ status.**

Line 66–68: Its application to the orthopaedics field is innovative, aiming to ascertain whether it can offer additional predictive value beyond the capabilities of CRP or the other parameters (NLR, PINI, NLR).

Line 68–75: Given the limitations of CRP as a standalone parameter to predict the IAC for the abovementioned reasons, we have hypothesized that various malnutrition or inflammatory parameters might offer a superior prediction of IAC during the preoperative and postoperative courses, and these parameters included ICIS and PINI that have not yet been investigated in orthopaedics. If these parameters prove reliable in predicting IAC, it will allow better identification of at-risk patients, leading to better pre- and postoperative care optimisation. A parameter meeting all of the requirements would significantly benefit clinical practice as it could reduce IAC and improve overall patient outcomes.

**Comments to the Authors:** I do not agree with non-implant associated complications such as thromboembolism, urinary tract infection and peroneal nerve paralysis being categorised under implant-associated complications. They are poorly related to the chosen predictors and would invalidate the results of the study.

Authors´ comment: Thank you for this commentary, but we cannot agree with it. All described IAC are associated with arthroplasty as far as IAC occurred in apparent association with arthroplasty in the early (6-7 weeks) postoperative period. We have adjusted the urinary tract infection to make it evident that it is mitigated.

Changes in the text:

Line 209–213: Mitigated urinary tract infections occurred in two patients (1.26%) and were treated using susceptible antibiotics without a negative impact on arthroplasty. Three (1.89%) patients suffered from thromboembolism after TKA or THA and were managed according to the internal hospital guidelines. One (0.63 %) patient with peroneal nerve paralysis after THA was taken in the care of a physiotherapist and underwent appropriate procedures.

Page 8: Table 1: Basic characteristics of patients, surgery, antibiotic prophylaxis and complications

**Comments to the Authors:** The authors stated there were twelve patients who developed implant-associated complications, including six SSIs, however this did not correspond to data presented in Table 1.

Authors´ comment: Thank you for observation. We have reviewed the Table 1.

Changes in the text: Correction of the Table 1:

Page 8: Table 1: Basic characteristics of patients, surgery, antibiotic prophylaxis and complications

**Comments to the Authors:** Interpretation of the data must be performed in the context of clearly stated hypothesis / aims. I am unable to provide specific comments without clarification of the study aims. Generally speaking, p-values alone are inadequate in terms of identifying significant results; the size of between-group difference and confidence intervals must also be taken into account. For example, the IAC negative group has a pre-operative albumin of 43.3 versus 39.6 in the IAC positive group with a p-value of 0.042. The size of the difference (less than 4 g/L) is small when compared with the SSI groups, where the values were 43.6 and 30.6 meaning a difference of 13 g/L between the SSI-positive and SSI-negative groups. The p-value was 0.076 which is above 0.05 due to the small sample size (n=6) in the SSI positive group.

Authors´ comment: Thank you for your insights and suggestions. We acknowledged the necessity of interpreting our data within the context of explicitly stated hypotheses and aims. In response to your comments, we agree that relying merely on p-values to discern significance can be inadequate. Moreover, the statistical test in our study did not have enough power to find a statistically significant difference. Therefore, the results must also be evaluated based on numerical differences between parameters and potential clinical significance.

In our analysis, the observed difference in pre-operative albumin levels between the IAC-negative (43.3 g/L) and IAC-positive (39.6 g/L) groups was 3.7 g/L with a p-value of 0.042. Although statistically significant, this difference is modest, particularly when compared to the 12.0 g/L difference observed between the SSI-positive and SSI-negative groups, despite a p-value of 0.076, which reflects the smaller sample size in the SSI-positive group (n=6).

This discrepancy highlights the critical distinction between statistical significance and clinical significance. Therefore, clinical significance relates to whether these findings have practical implications in a real-world setting, which, in our case, suggests a need for cautious interpretation, as we indicate in the conclusion section. We have edited the interpretation of the results accordingly.

Moreover, your feedback is more or less related to the Reviewer 3 commentary – sample size calculation and estimated effect size; therefore, see the relevant response to the Reviewer 3 commentary. Furthermore, it should be noted that this response was consulted with a statistician.

Changes in the text:

Line 272–275: A limiting factor was the small sample size, which produced low outcomes, limiting the possibility of significant statistical results for the evaluated parameters **with enough power to detect statistically significant differences. However, even when there is a statistically significant difference, the result should be interpreted cautiously, especially in clinical practice**

Line 300–302: Further research with larger cohorts is warranted to substantiate these observations, especially their clinical significance.

**Comments to the Authors:** Please update reference source on page 6 line 138

Authors´ comment: Thank you for this insight. We have revised all references sources in the document.

Changes in the text: Reference source for Table 1 has been updated.
